# Supplementary material for: Deficiency of PRKD2 triggers hyperinsulinemia and metabolic disorders
Source: Nat Commun. 2018 May 22;9:2015. doi: 10.1038/s41467-018-04352-z (PMC5964083; doi:10.1038/s41467-018-04352-z)
Supplement: Supplementary file 1 — Supplementary Information [file 41467_2018_4352_MOESM1_ESM.pdf]

Supplementary Information

Deficiency of *PRKD2* Triggers Hyperinsulinemia and Metabolic Disorders

Xiao et al.

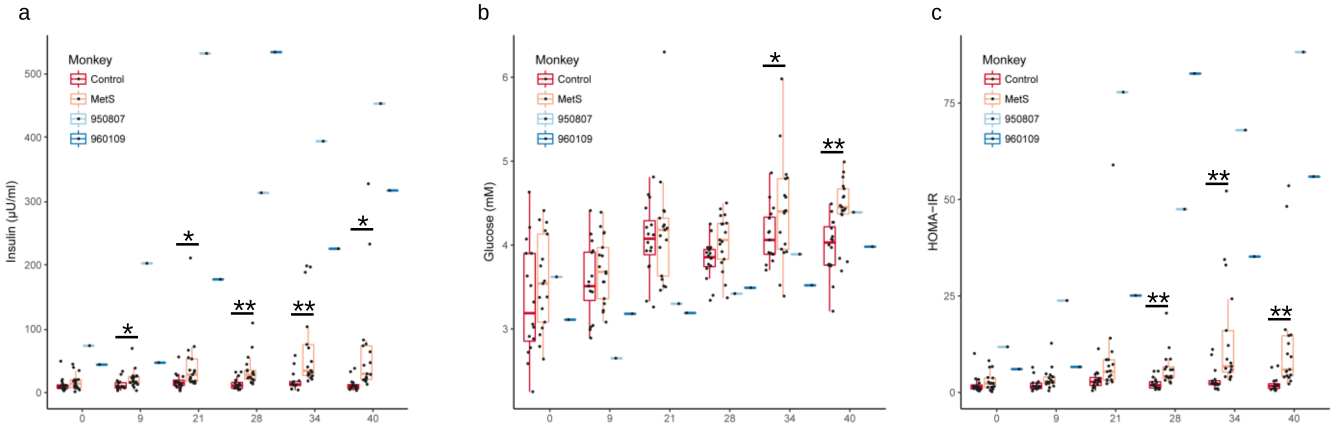

Supplementary Fig. 1 Changes of glucose and insulin levels in hyperinsulinemic monkeys (dot plots and box plots). **a** Fasting plasma insulin levels of control, MetS, and hyperinsulinemic monkeys in follow-up studies. **b** Fasting plasma glucose levels of control, MetS, and hyperinsulinemic monkeys in follow-up studies. **c** Homeostasis model assessment of insulin resistance (HOMA-IR) of control, MetS, and hyperinsulinemic monkeys in follow-up studies. *p <0.05, **p <0.01, ***p <0.001, control vs MetS (Control, n=16; MetS, n=17). All data are represented as mean ± SEM, the significant difference between groups was assessed by the Student's *t*-test.

Supplementary Fig. 2

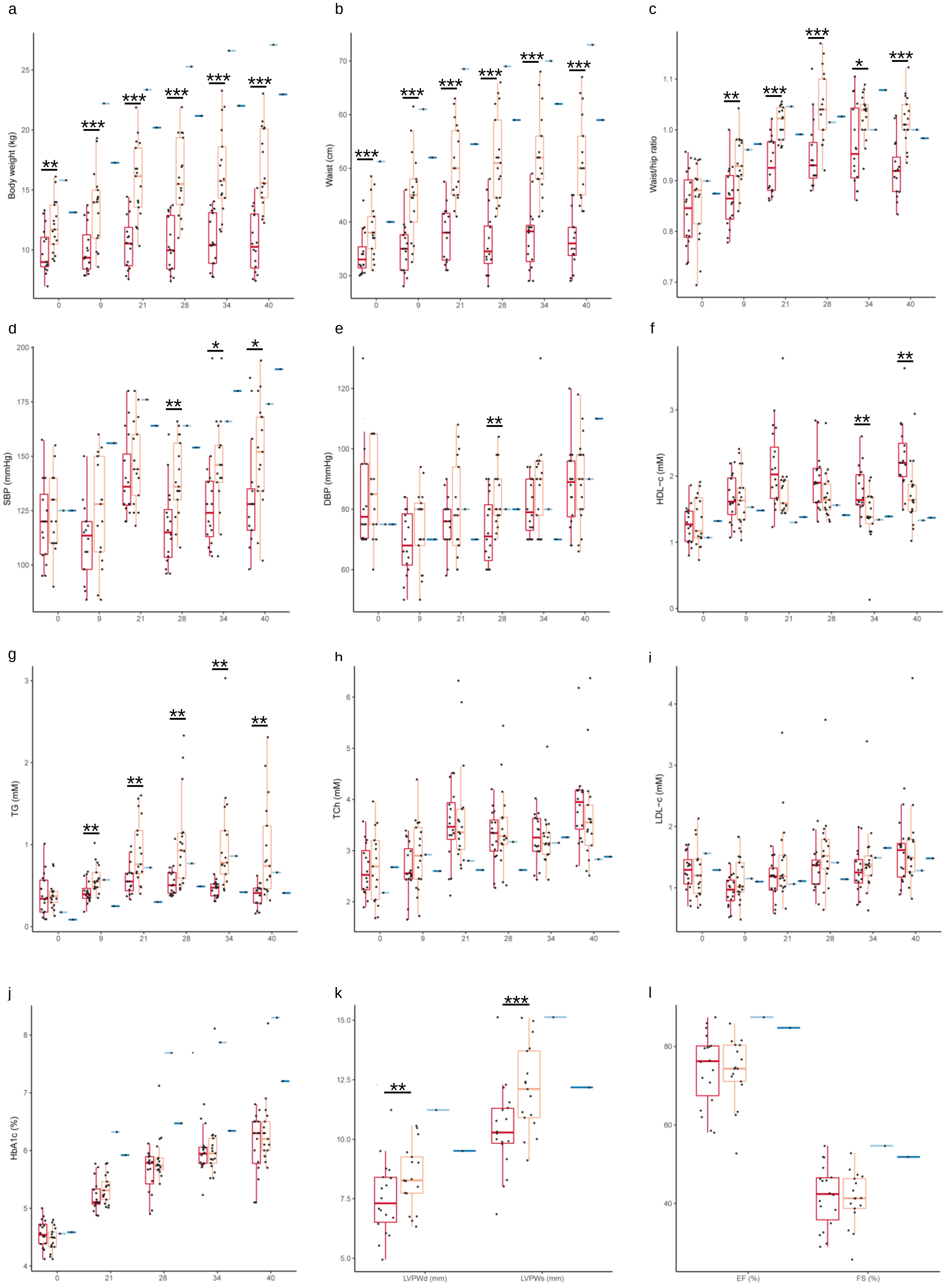

Supplementary Fig. 2 Metabolic and anthropometric parameters of control, MetS, and hyperinsulinemic monkeys (dot plots and box plots). Related to Fig 2. **a** Body weights. **b** Waist circumferences. **c** Waist/hip ratios. **d** SBP, systolic blood pressures. **e** DBP, diastolic blood pressures. **f** HDL-c, high-density lipoprotein cholesterol. **g** TG, triglyceride. **h** TCh, total cholesterol. **i** LDL-c, low-density lipoprotein cholesterol. **j** HbA1c, hemoglobin A1c. **k** LVPWd (left ventricular posterior wall thickness at end-diastole) and LVPWs (left ventricular posterior wall thickness at end-systole) at 40 months of follow-up studies. **l** EF (ejection fraction) and FS (fraction shortening) at 40 months of follow-up studies. *p <0.05, **p <0.01, ***p <0.001, control *vs* MetS (Control, n=16; MetS, n=17). All data are represented as mean ± SEM, the significant difference between groups was assessed by the Student's *t*-test.

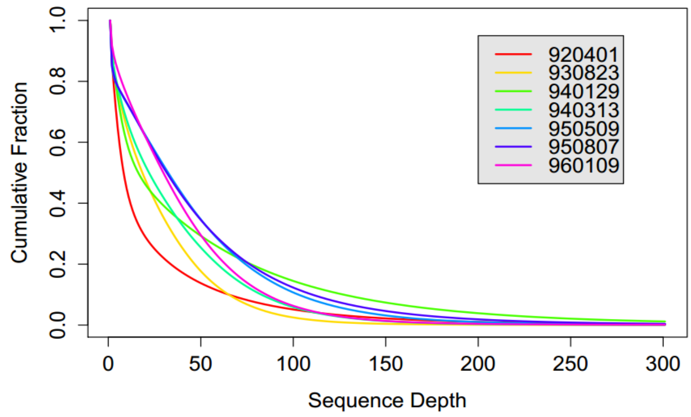

Supplementary Fig. 3 Statistics for exome-seq. Related to Fig 3. For each dataset, the sequencing depth is summarized and shown in cumulative frequency plot.

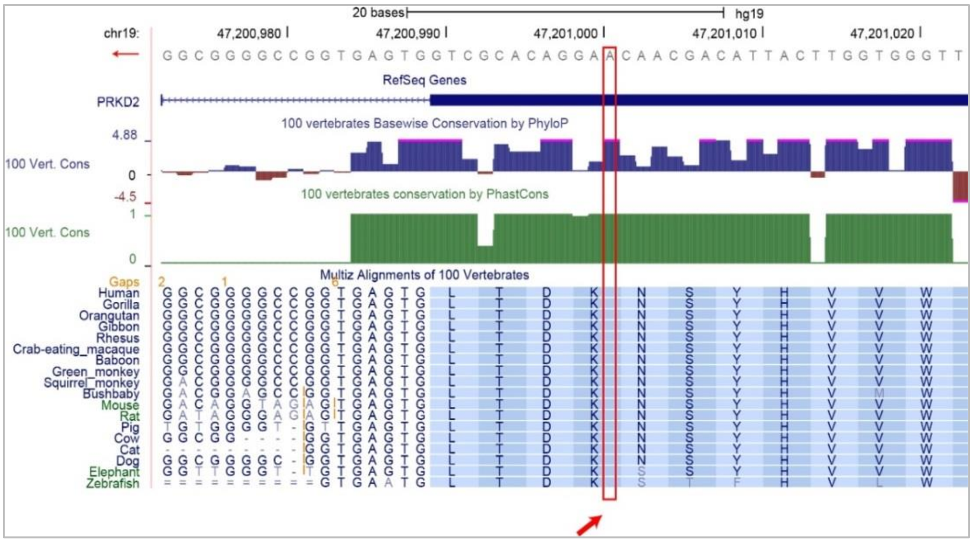

Supplementary Fig. 4 The conserved *PRKD2* K410 site. Related to Fig 3. A UCSC genome browser snapshot showing the aligned sequences of *PRKD2* across multiple species, with the position of the K410X nonsense mutation highlighted in red rectangle. The conservation levels of these sites are measured by *Phylop* and *PhastCons*.

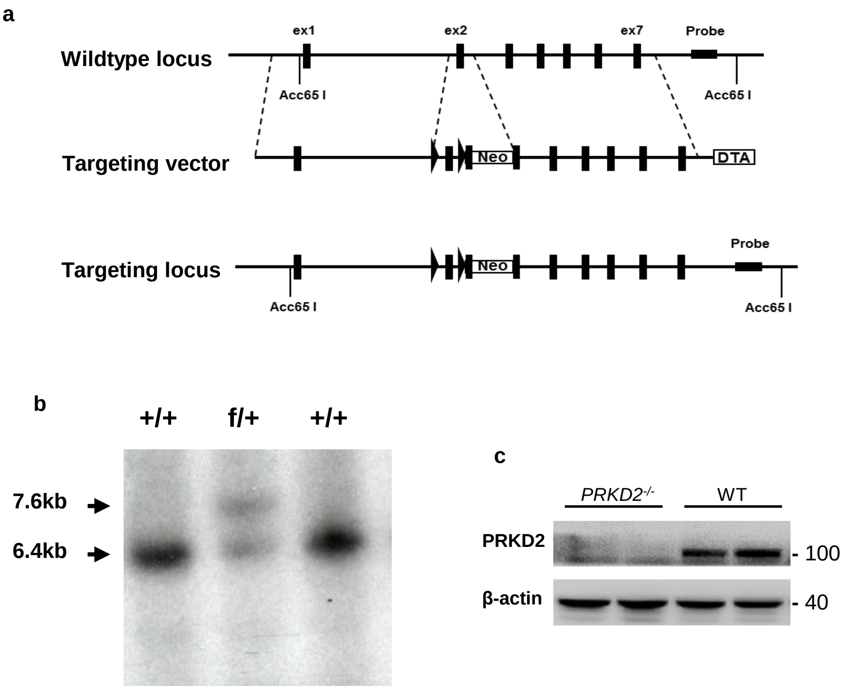

Supplementary Fig. 5 Generation of global *PRKD2* deficient mice. Related to Fig. 4, 5 and 6.

a Targeting strategies for mouse *PRKD2* gene. A restriction map of the relevant genomic region (top), the targeting vector (middle) and the targeted locus after recombination (bottom) is shown. The targeting construct was generated by flanking exon 2 of *PRKD2* with loxP sites, while frt sites flank the Neo-cassette. Neo represents the neomycin resistance gene; the arrowheads represent LoxP sites and the long boxes represent frt sites. **b** Detection of wild type (+/+) and targeted allele (f/+) for the *PRKD2* gene by DNA Southern blot analysis. The 7.6- and 6.4-kb bands represent the WT and targeted alleles, respectively. **c** Representative western blot of *PRKD2* protein levels in liver tissue of *PRKD2-/-* and WT mice.

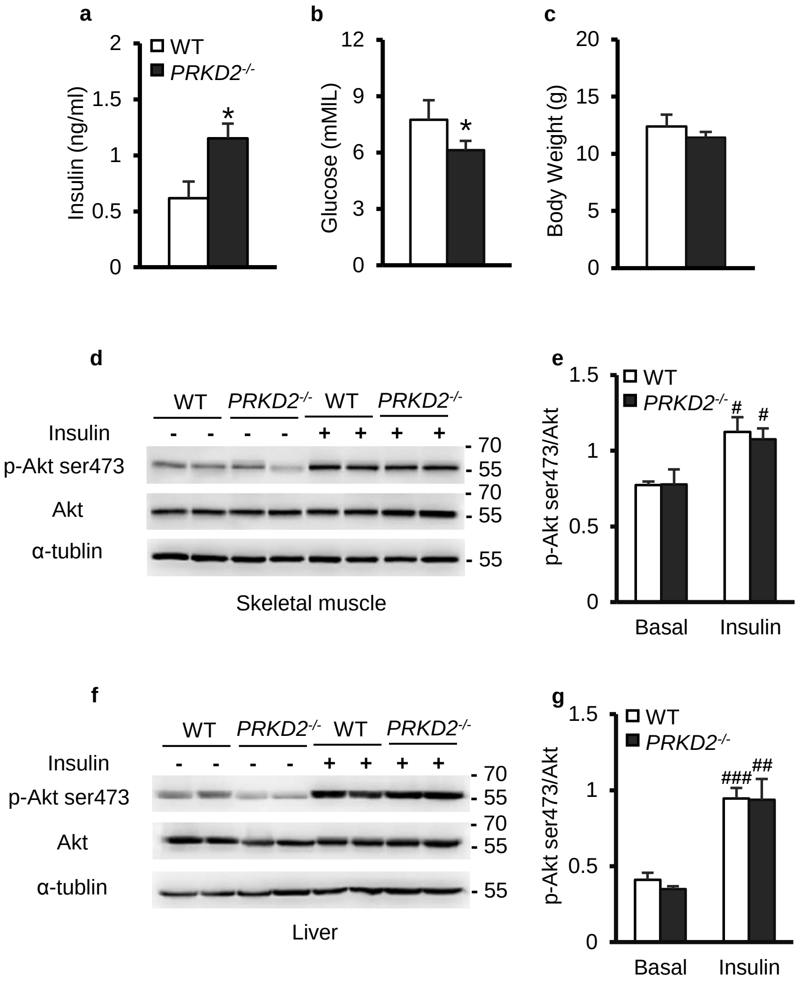

Supplementary Fig. 6 *PRKD2* deficiency enhanced Insulin secretion in 4-week old mice. Related to Fig. 4 and Supplementary Fig. 5. **a** Fasting serum insulin in WT and *PRKD2-/-* mice, **p* <0.05, WT *vs PRKD2-/-* (WT, n=5; *PRKD2-/-*, n=7). **b** and **c** Fasting blood glucose (**b**) and body weights (**c**) in WT and *PRKD2-/-* mice, **p* <0.05, WT *vs PRKD2-/-* (WT, n=7; *PRKD2-/-*, n=9). **d** and **e** Representative western blot (**d**) and statistics of Akt phosphorylation (**e**) in skeletal muscle of WT and *PRKD2-/-* mice. **f** and **g** Representative western blot (**f**) and statistics of Akt phosphorylation (**g**) in liver of WT and *PRKD2-/-* mice #*p* <0.05, ##*p* <0.01, ###*p* <0.001, basal *vs* insulin (Basal: WT, n=4; *PRKD2-/-*, n=7; Insulin: WT, n=6; *PRKD2-/-*, n=8). All data are represented as mean ± SEM, the significant difference between groups was assessed by the Student's *t*-test.

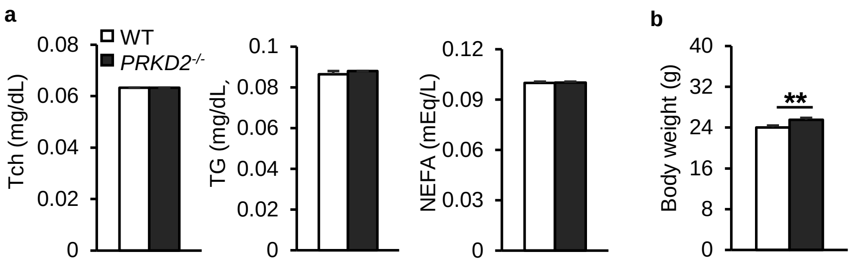

Supplementary Fig. 7 *PRKD2* deficient increses body weight in 14-week old mice. Related to Fig 4. **a** Blood lipid profiles of *PRKD2-/-* and WT mice (WT, n=24; *PRKD2-/-*, n=27). **b** Body weights of *PRKD2-/-* and WT mice. ***p* <0.01, WT *vs PRKD2-/-* (WT, n=50; *PRKD2-/-*, n=48). Data are represented as mean ± SEM, the significant difference between groups was assessed by the Student's *t*-test.

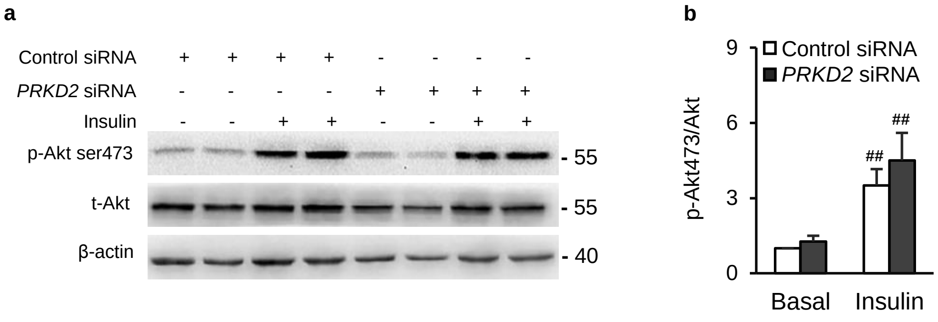

Supplementary Fig. 8 *PRKD2* knocking down do not influence insulin signaling pathway in Hepa1-6 cells. Related Fig. 5. **a-b** Western blot of Akt ser473 and total Akt in *PRKD2*-knockdown cells (**a**) and statistics (**b**), # # p<0.01, basal *vs* insulin (n = 8/group). Data are represented as mean ± SEM, the significant difference between groups was assessed by the Student's *t*-test.

Supplementary Fig. 9 Uncropped western blots for figures in this study.

Uncropped western blots for Fig. 3d

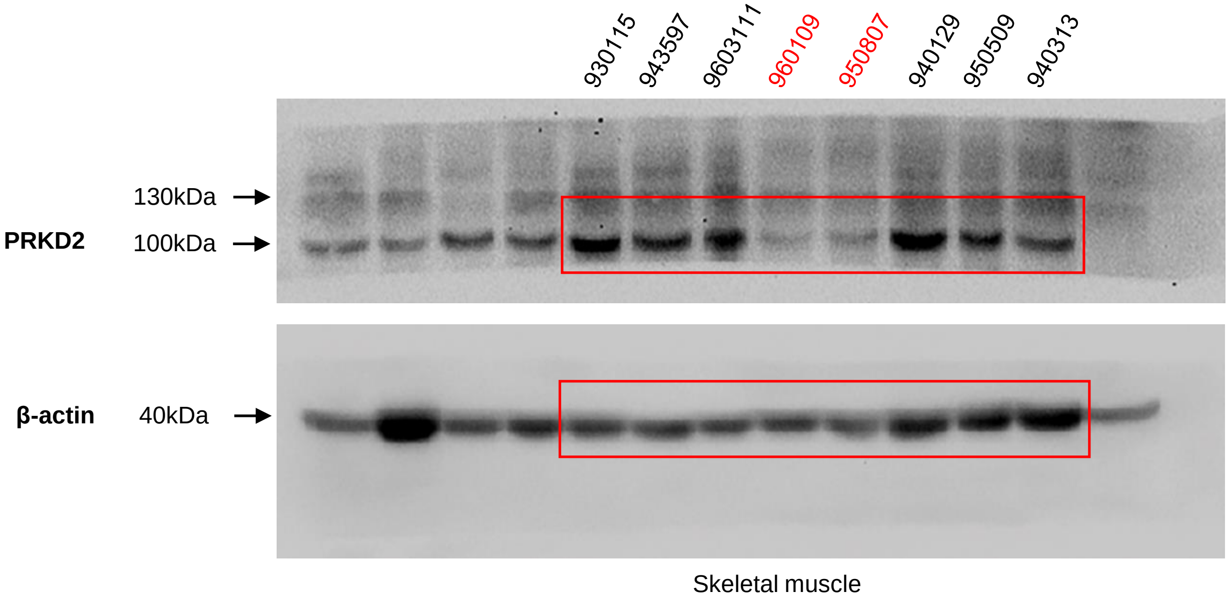

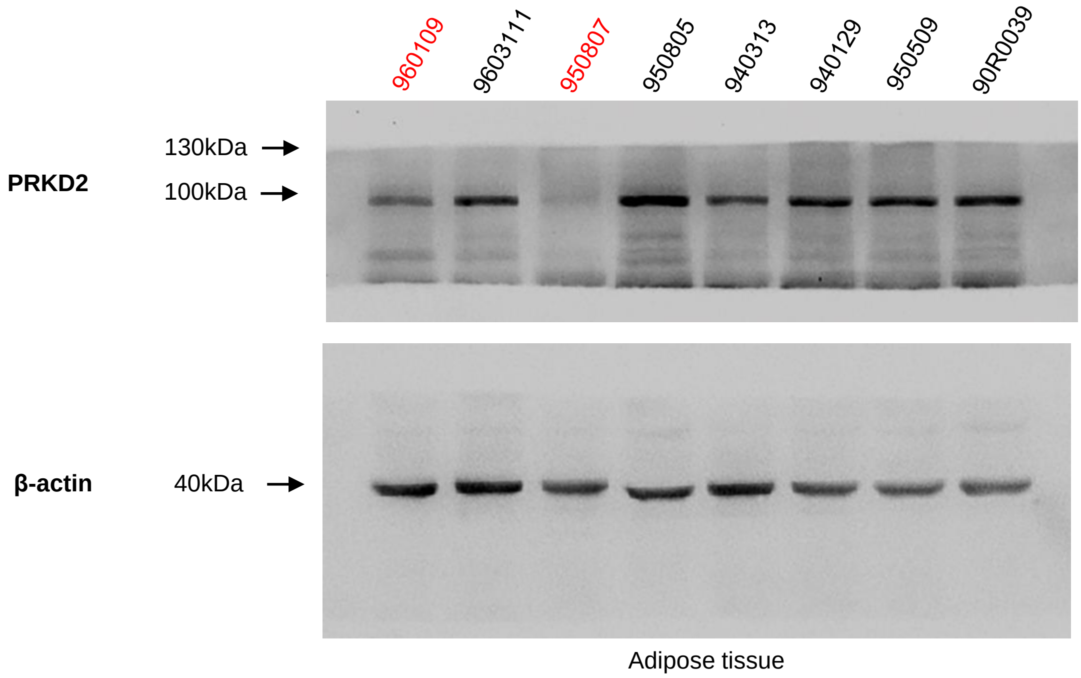

Uncropped western blots for Fig. 4e

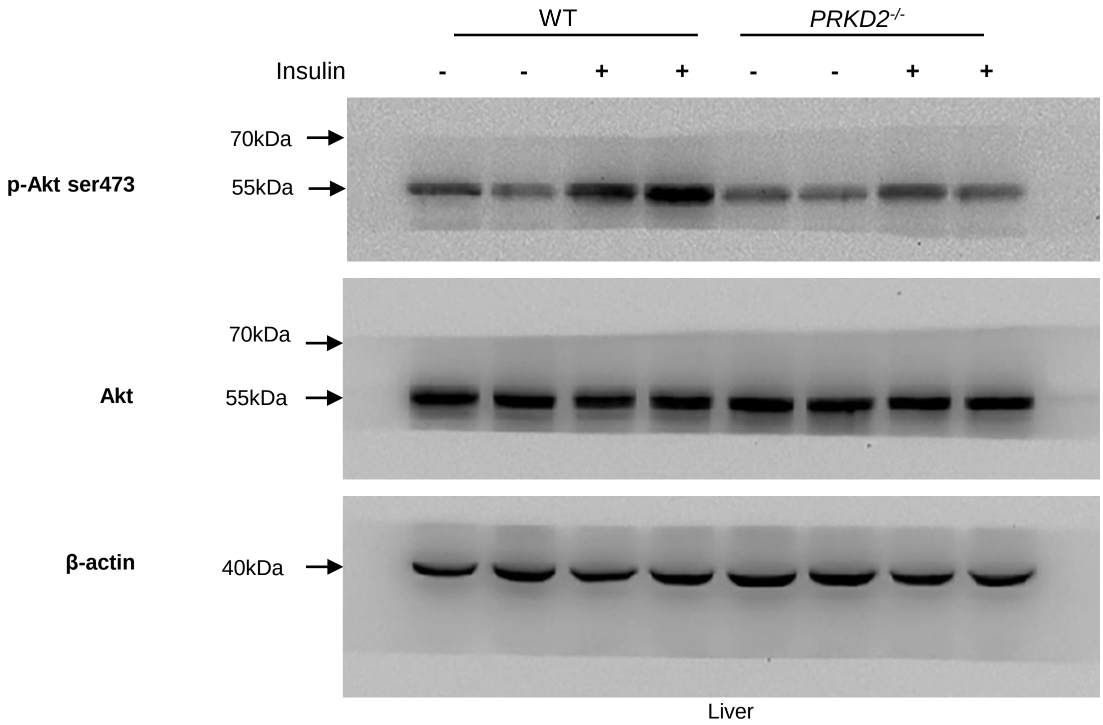

Uncropped western blots for Fig. 4g

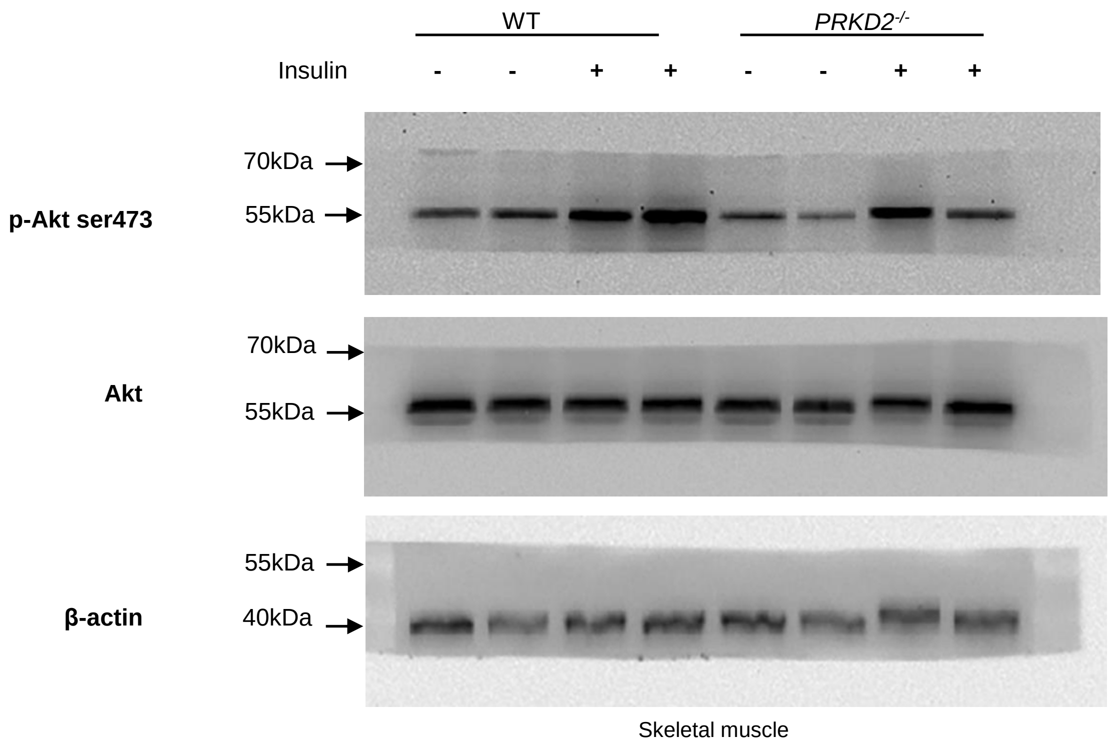

Uncropped western blots for Fig. 5g

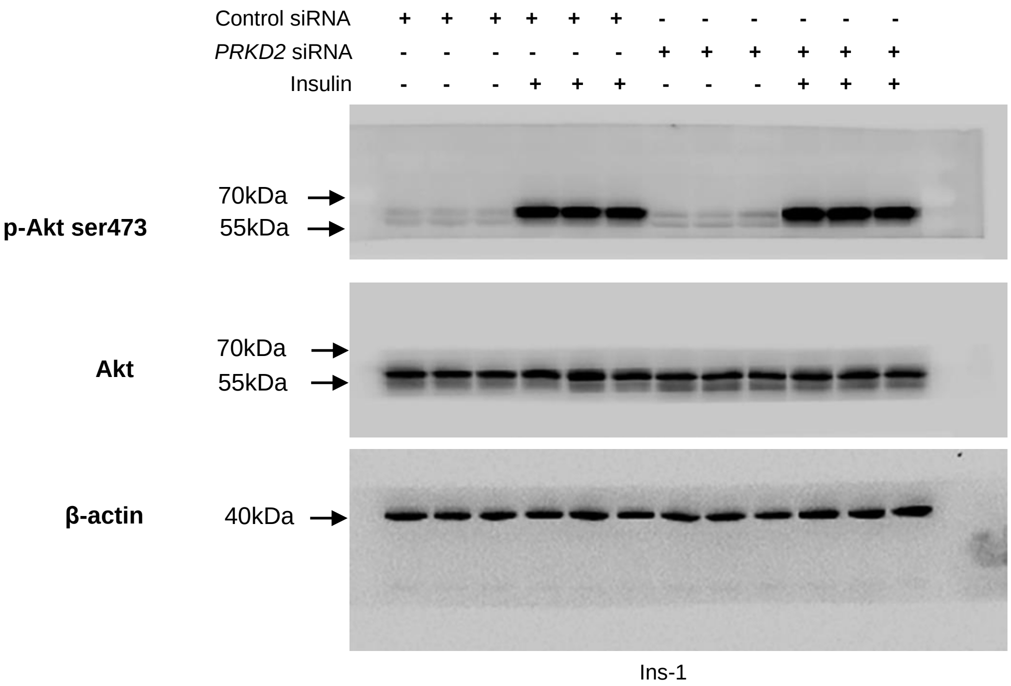

Uncropped western blots for Supplementary Fig. 5c

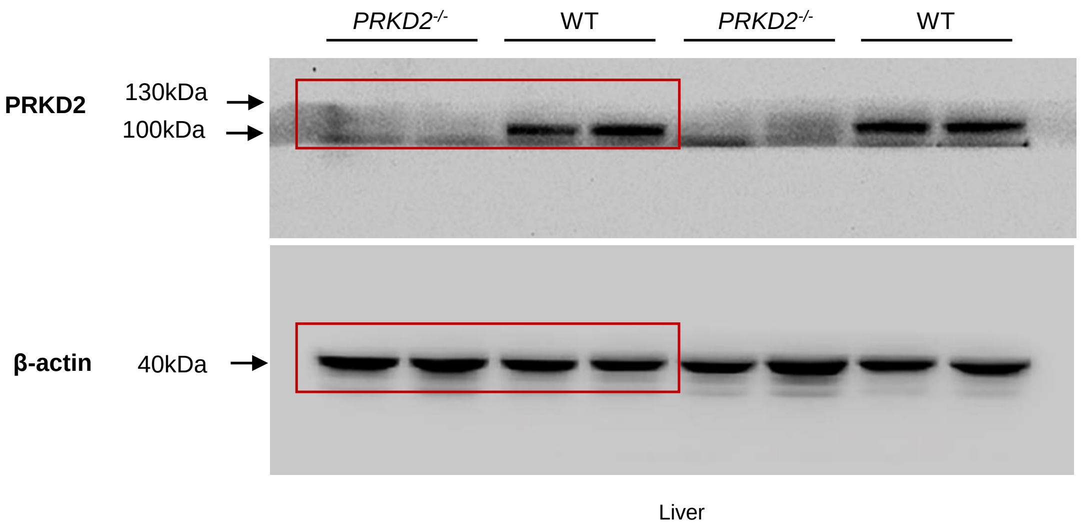

Uncropped western blots for Supplementary Fig. 6d

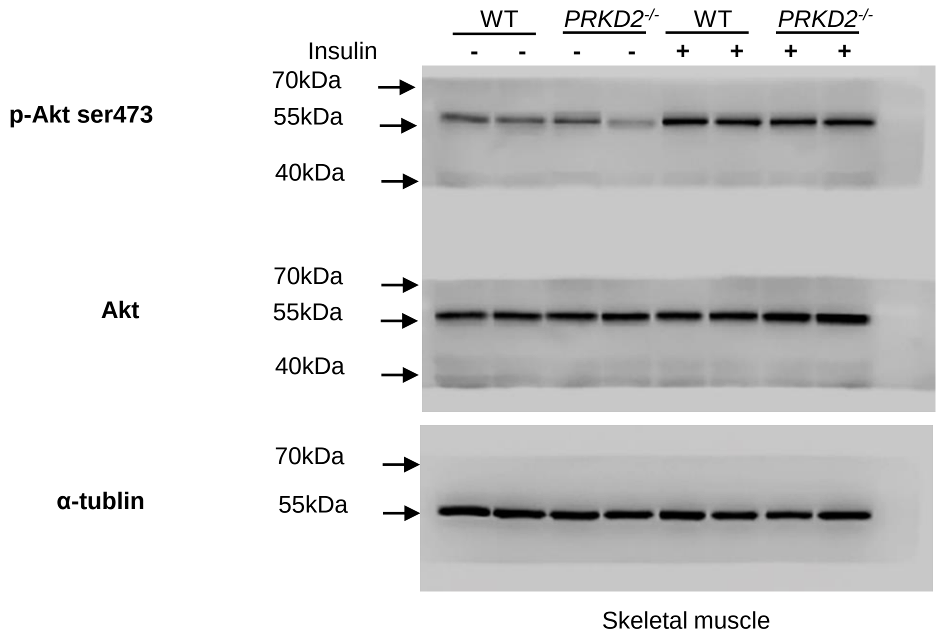

Uncropped western blots for Supplementary Fig. 6f

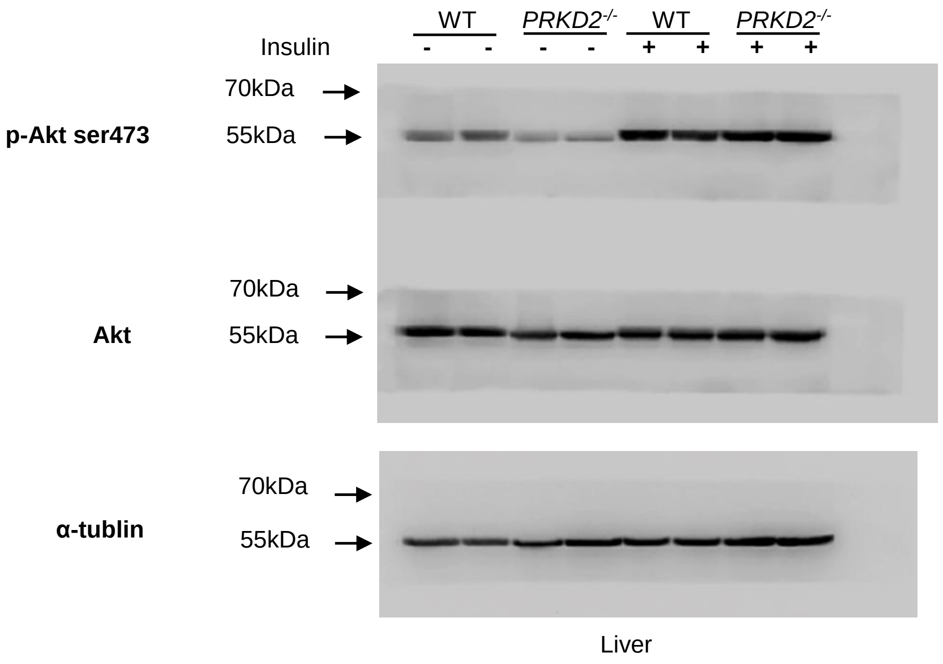

Uncropped western blots for Supplementary Fig. 8a

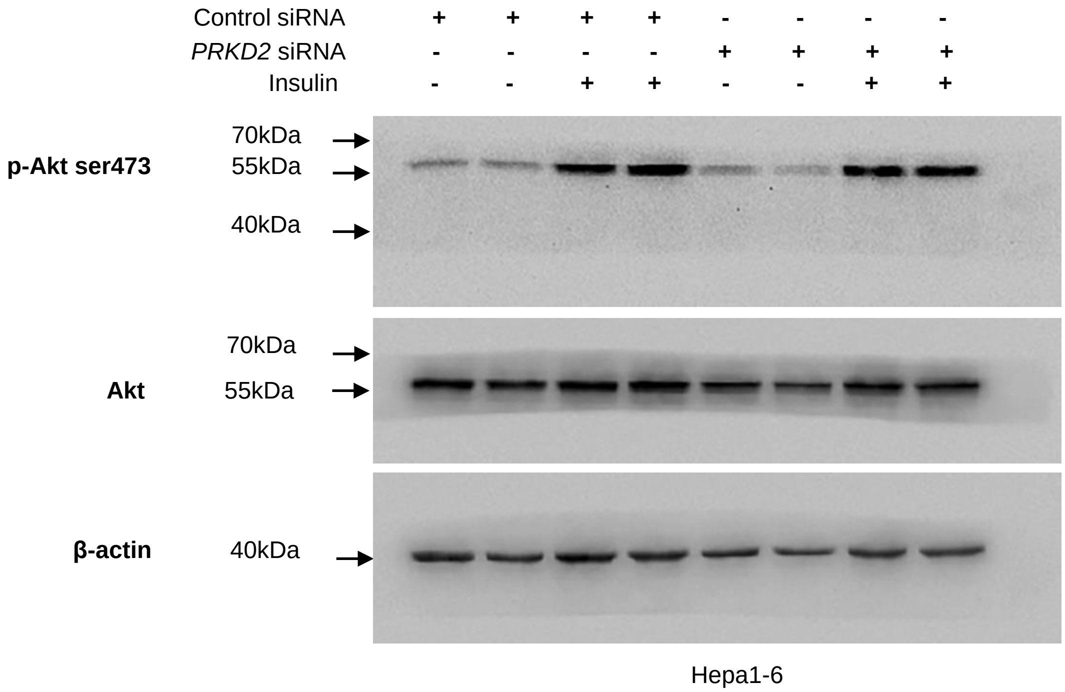

Supplementary Table 1. Statistics for exome-seq data. Related to Fig 3.

Sample	Total Reads	Length	Total Nucleotide	Q20	Uniquely-mapped Reads
**920401**	27,761,610	90	2,485,561,420	0.8648	18,019,560 (0.6490)
**930823**	25,419,728	90	2,276,178,280	0.9510	21,737,042 (0.8551)
**940129**	48,468,874	90	4,342,827,801	0.8969	35,470,438 (0.7318)
**940313**	32,013,296	90	2,868,599,738	0.9197	26,978,396 (0.8427)
**950509**	37,938,054	90	3,370,004,232	0.8968	31,026,458 (0.8178)
**950807**	38,758,796	90	3,392,475,085	0.8861	30,595,187 (0.7894)
**960109**	35,730,802	90	3,203,755,851	0.9347	29,894,585 (0.8367)
**Sum**	246,091,160		21,939,402,407		193,721,666 (0.7872)

Supplementary Table 2. Identification of candidate genetic variations underpinning hyperinsulinemia in the rhesus monkey MetS cohort. Related to Fig 3 and Supplementary Fig 1

SNP Category	Filter			
	(1) All SNPs in Target Region*	(2) Not in 5 Controls (950807 / 960109)	(3) Shared by 2 Cases	(4) Not in Public WGS Datasets#
**Nonsense**	785	21 / 12	2	1
**Frame-shift**	756	18 / 12	3	0
**Splice Site**	676	8 / 6	1	0
**Non-synonymous**	49,968	1,018 / 1,079	163	28
**Sum**	52,185	1,065 / 1,109	169	29
